# Vulnerability of fishery-based livelihoods to climate variability and change in a tropical island: insights from small-scale fishers in Seychelles

**DOI:** 10.1007/s43621-021-00057-4

**Published:** 2021-11-08

**Authors:** Daniel Etongo, Lyn Arrisol

**Affiliations:** 1 James Michel Blue Economy Research Institute, University of Seychelles, Anse Royale, Seychelles; 2 Department of Environmental Sciences, University of Seychelles, Anse Royale, Seychelles

**Keywords:** Seychelles, Climate variability, Climate change, Livelihoods, Index, Fisheries

## Abstract

While climate shocks and stressors are not unique to developing countries, their impacts are expected to be most acute here due to limitations in the assets and infrastructure required for adaptation. This study assesses the vulnerability of fishery-based livelihoods to the impacts of climate variability and change across two major islands in Seychelles based on 80 household surveys and three shared dialogue workshops (SDWs) with small-scale fishers. Results showed that the percentage of fishers’ households that depend mainly on fisheries as a source of income was 95% and 97% for Mahe and Praslin respectively with alternative income streams along the fishing value chain such as transportation, fish mongers and processor. Fishers on Mahe Island had a dependency ratio index that was slightly higher than those on Praslin. Overall, fishing households on Mahe showed greater vulnerability on socio-demographic profile index compared to their counterparts on Praslin. However, greater livelihood diversification was recorded for householders on Mahe than Praslin as fishers earned income from tourism-related activities such as guest house, car rental, boat ride, and sales of coconuts as mentioned during the SDWs. Fishers on Mahe struggled to find fish for 3 months during the southeast monsoon season compared to 2 months for those on Praslin given that access is granted to fish in the lagoon during this season. More importantly, the voluntary closure of some fishing zones between the months of November to April on Praslin is a sustainability strategy that witnessed an increase in size and number for both rabbit and parrotfish. Further studies are needed in two key areas as follows: the role of subsidies and sustainable fisheries management, and a value-chain approach to vulnerability of small-scale fishers within the fishery sector in Seychelles.

## Introduction

Small-scale fisheries (SSFs) account for more than 90% of the world’s capture fishers and provide livelihoods and food security for millions of individuals around the globe [1, 2]. In coastal communities especially in developing countries and most Small Island Developing States (SIDS), SSFs are characterised by labour-intensive harvesting strategies, relatively small fishing vessels, short fishing trips, close to shore, low relative catch per vessel and limited capital investment [3]. This sector also faces major challenges including lack of access to financing, restricted market access, the need to ensure quality of fish products, diseconomies of scale and not being able to add value to their catches [4]. Therefore, small-scale fishing communities are amongst the most destitute socio-economic groups in developing countries. Small-scale fisheries are exposed to a range of natural and anthropogenic stressors, such as growing competition for natural resource, economic and financial uncertainty and policy that require changes in their behaviour to mitigate damages and to take advantage of opportunities [5, 6]. Climate change is another stressor that affects food security and employment especially those livelihood activities considered to be climate sensitive by the Intergovernmental Panel on Climate Change (IPCC) such as agriculture, fisheries and tourism [7–11]. Its impacts such as rising global average temperature and changes in precipitation patterns are already affecting ecosystems, biodiversity and human systems throughout the world [5, 8] and across sub-Saharan Africa based on recent study in the sub-region [12]. According to Barange et al. [13], loss of production and infrastructure arising from the increased occurrence of extreme weather events such as floods, cyclones and droughts are some of the short-term climate change impacts on SSFs.

Several studies argued that oceans and seas are the most affected by the process of change caused by global warming as they represent a large portion of our planet [14–19]. As such, island vulnerability to high tides, floods, sea level rise and coastal erosion which according to Westlund et al. [20], are likely to contribute towards the degradation of land-based infrastructures such as landing sites, thereby leading to disruption in fishing activities. While climate shocks and stressors are not unique to developing countries, their impacts are expected to be most acute here due to limitations in the assets and infrastructure required for adaptation that in turn limit the optimal performance of the fishery sector in enhancing people’ livelihoods [21–26]. While few positive impacts on fisheries have also been reported, such as increased nutrient production in high latitude [26], seasonal increase in growth of rainbow trout [27], and reduced cold-water mortalities of some aquatic animals, most of the impacts of climate change are overwhelmingly negative [28]. For example, climate-driven reductions in fisheries production and alterations in fish-species composition will subsequently increase the vulnerability of tropical countries with limited adaptive capacity [29]. Climate change will tend to exacerbate non-climatic pressures on fisheries such as overfishing, pollution, and loss of habitat [30]. Increasing temperatures, altered precipitation patterns, sea level rise, ocean acidification, and changes in dissolved oxygen concentration all affect the structure and productivity of marine and coastal ecosystems and fish populations [28, 31]. These impacts have already extended to fishery-dependent communities especially among small-scale fishers as documented in a study conducted in Bangladesh [32].

Small-scale fisheries encompassing all activities along the value chain—pre-harvest, harvest and post-harvest—undertaken by men and women, and play an important role in food security and nutrition, poverty eradication, equitable development and sustainable resource utilization [33]. Despite some difficulties for a distinction between SSFs and “large-scale” or “industrial” fisheries, typically, the division hinges on assumptions about the role of fishing technologies and the nature of human progress [34]. For example, SSFs is firmly rooted in the traditions and values of local communities with many small-scale fishers that usually provide fish for direct consumption within their households or communities and also for sale to generate income [35]. An estimated 90% of all people directly dependent on capture fisheries work in the small-scale fisheries sector. As such, small-scale fisheries serve as an economic and social engine, providing food and nutrition security, employment and other multiplier effects to local economies and livelihoods especially in SIDS [35]. Among its many targets, SDG14b speaks directly to small-scale fisheries, calling for secured access to resources and markets for this sector [36] and the alignment of the SDG targets with the Voluntary Guidelines for Securing Sustainable Small-Scale Fisheries [35]. However, such an alignment does not entirely guarantee sustainability of small-scale fisheries [33], because aside from an integrated approach that addresses co-benefits and trade-offs [37, 38], the impacts of climate variability and change cannot be ignored.

In Seychelles, the small-scale fishery consists of artisanal and semi-industrial fishing with an estimated 2–3% from both subsectors as contributions to the annual Gross Domestic Product [39]. The total fish catch for the year 2019 was 1703 tonnes compared to 2427 tonnes in 2018, which correspond to a decrease of 30% [39]. According to King and Nageon de Lestang [40], the fishery sector is an essential source of revenue, employment, food security, and foreign exchange in the Seychelles given that land-based activities such as agriculture and large-scale industrial activities are constraint by the relatively small land size of 455 km^2^ of which 50% of the land area is protected [41]. Approximately 17% of the total population is employed in the fishery sector and those that are directly dependent on small-scale fisheries correspond to 30% of the population while another 10% of the population benefits or are indirectly dependent on this sector with tourism as a Blue Economy sector contributing 65.8% in 2019 and 61% in 2020 to its GDP [42]. Seychelles, just like other SIDS depends largely on its Blue Economy for livelihood benefits and its national development which is also not immune to the impacts of climate change and those that depends on it for food and other livelihood values are exposed to varied levels of vulnerabilities.

For example, coral bleaching events are becoming more frequent and severe. The Western Indian Ocean was affected by the 1998 coral bleaching episode during which the Seychelles lost over 90% of its original coral cover [43]. It’s important to reiterate that some coral reef fish are dependent on live coral, feeding directly on it, using it as refuge from predators or as a site for settlement of their larvae. These species, belonging to the fish family *Monaenthids*, *Chaetodontids* and *Pomacentrids*, that are also the principal target species for reef fishery, often decline rapidly in health and abundance following coral mortality as was the case in Seychelles [44]. Aside from the 1998 coral bleaching event that greatly affected the fishery sector and the economy in Seychelles, another coral bleaching event occurred in 2016 between the months of February to August [45]. A particularly austral hot summer coupled with an intense positive Indian Ocean Dipole and an El Niño event caused water temperatures to rise and exceed seasonal averages by 1 to 2 °C for several weeks, triggering the phenomenon of coral bleaching on the reef [45]. Besides the challenges posed by coral bleaching, the fishery sector is also highly susceptible and vulnerable to the rapid social and economic effects of the COVID-19 pandemic [46]. The federation of associations regrouping artisanal fishers from Mauritius, Comoros, Madagascar, and Seychelles recently launched a study to evaluate the impact of COVID-19 on their activities in the Indian Ocean Region [47]. The chairperson of the federation during the launch of the project said “each country has its own specificity and although the challenges might be the same they can also differ in both nature and intensity, which is why to get a very clear picture”. The Chairperson speaking specifically to the situation in Seychelles further mentioned that “like other industries when the pandemic hit we felt the adverse effect. With tourism on the brink, we also lost the biggest bulk market for our products, as hotels used to buy large quantities of fish for their restaurant needs. With less tourists in the country, the demand for our fish fell, while with the fall in the value of the rupee, our operational costs skyrocketed” [47].

Examining the vulnerability of fishing communities and householders to climate variability and change can help identify and characterise actions that can mitigate adverse impacts. Despite the importance of small-scale fisheries, knowledge of climate-induced impacts and vulnerability at the household or community scale remains limited with few studies across coastal communities as highlighted in a recent global systematic review [48]. More importantly, many studies on vulnerability assessment depend on large-scale and top-down research using predetermined indicators that make implicit assumptions about the nature of changes being experienced. Therefore, our study addresses this knowledge gap through the development of locally sensitive indicators which is important for informing effective interventions. Additionally, even though small-scale fishery are important for employment and food security especially for SIDS, knowledge on the vulnerability of fishery-based livelihoods to climate variability and change is scant and to the best of our knowledge this is the very first study that addresses this topic in Seychelles. The objective of this study is to assess the vulnerability of fishery-based livelihoods to the impacts of climate variability and change on small-scale fishing households on Mahe and Praslin Island. Local factors that affects vulnerability among small-scale fishing households such as socio-demographic characteristics e.g. number of fishing years, household dependency ratio, to economic, livelihood options and institutional factors were considered across the three major categories often used in vulnerability assessment—exposure, adaptive capacity and sensitivity. Therefore, this research can contribute to the management strategies at community level that can reduce the impacts of climate change by improving the adaptive capacities of small-scale fishers through livelihood diversification. More importantly, it will be of benefit to the Seychelles Conservation and Climate Adaptation Trust (SeyCCAT) whose main mandate is to strategically invest in ocean stakeholders to generate new learning, bold actions and sustainable blue prosperity in Seychelles.

## Vulnerability assessment: key concepts and conceptual framework

**Fig. 1:**
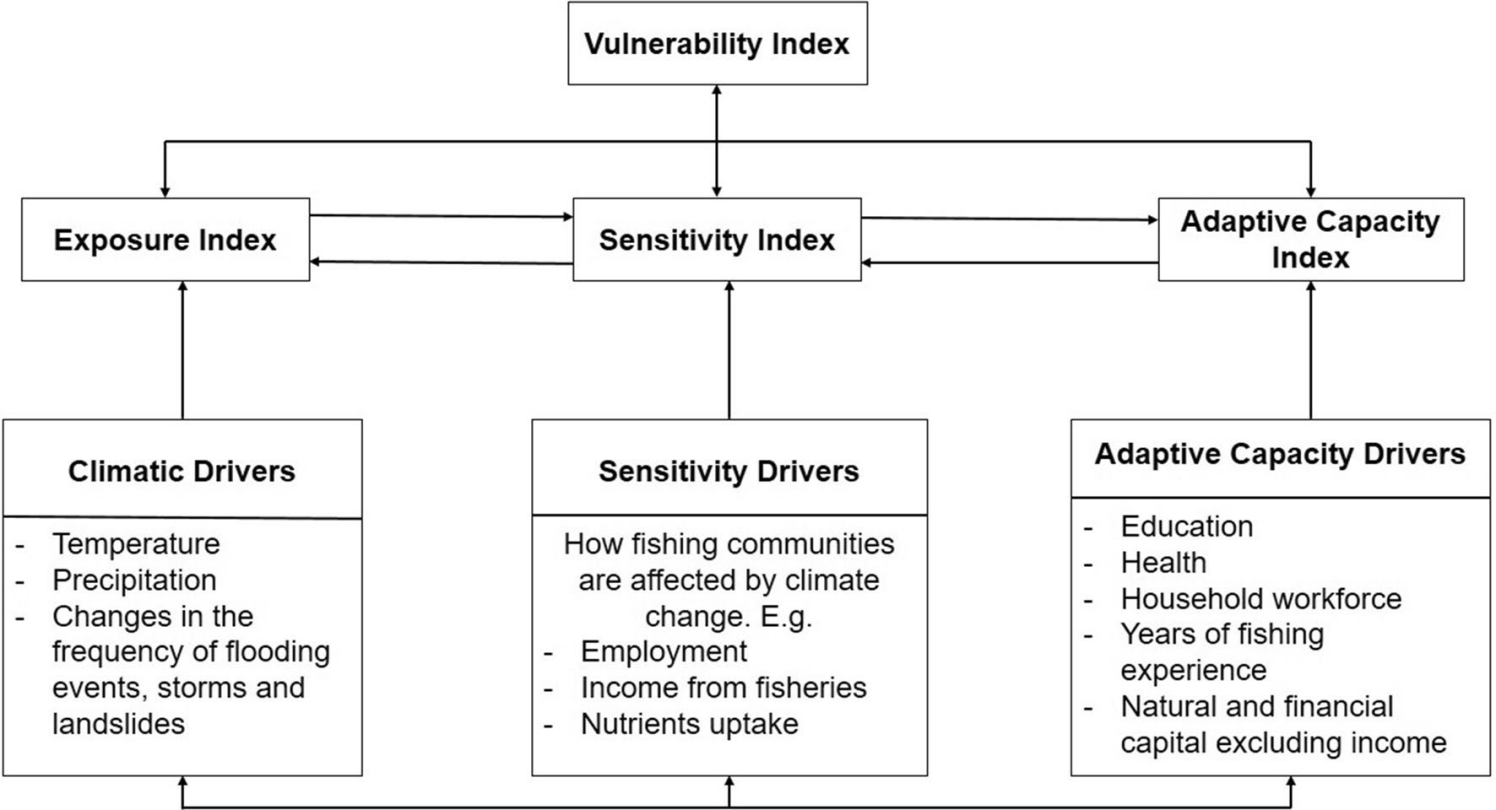
Schematic representation of the vulnerability conceptual framework. Source: Authors’ conceptualization

The concept of vulnerability is crucial and key in the discourse on development and livelihoods [49], hazards [50], global environmental change [32] and resilience thinking [51]. Firstly, vulnerability could be viewed as an outcome, hence the “end point’’ vulnerability analysis is to estimate and reduce costs of a hazard. Secondly, vulnerability is regarded as the ‘‘starting point’’ which focuses on the historical factors or current characteristics of individuals, households, communities, sectors, nations that is used to determine their differential susceptibility to harm [52]. On the other hand, the degree to which trends and shocks, driven by changes at various scales are experienced by resource groups in a region – small-scale fishers in the case of Seychelles, is referred to as exposure. Figure 1 provides a conceptual framework on the vulnerability of fishery-based livelihoods to the impacts of climate change highlighting the interaction between exposure, sensitivity and adaptive capacity in the context of Seychelles.

Furthermore, the susceptibility of a system to the effects of an exposure is sensitivity which is determined by historical, social, political, economic and environmental preconditions. Local factors have also been shown to affect vulnerability at the community level among small scale fishers. Islam et al. [32] in their study in Bangladesh described how the most exposed community is not necessarily the most sensitive or least able to adapt because livelihood vulnerability is a result of combined but unequal influences of biophysical and socio-economic characteristics of communities and households. They went further to state that within a fishing community, where households are similarly exposed, higher sensitivity and lower adaptive capacity combine to create higher vulnerability. This and other empirical studies highlights the need to account for variability in social processes influencing vulnerability to climate change.

### Climate change vulnerability assessment

Climate vulnerability assessment has emerged to address the need to quantify how communities will adapt to changing environmental conditions and methodological insights have been provided based on an index/indicator approach in order to bridge the gap between the social, natural, and physical sciences [53]. The IPCC working definition is the most widely used and considers vulnerability as a function of exposure, sensitivity, and adaptive capacity [54]. Studies relying on secondary data have to structure their analytical framework around available data and some of the challenges are inconsistencies or missing data, and sometimes must combine data collected at different temporal or spatial scales [55, 56]. However, a combination of individual indicators and aggregate indices have been derived through expert judgments or principal component analysis within the framework of the composite livelihood vulnerability index and this method has been instrumental in vulnerability assessment [32, 57]. Many studies on vulnerability assessment depend on large-scale and top-down research using predetermined indicators that make implicit assumptions about the nature of changes being experienced. More empirical research are needed to ground theoretical work in the complexities of local experiences of multiple exposures [58, 59] and to better identify actions to improve institutions and policies [60] which is the focus of the current study that applies an indicator approach derived from expert judgement. The shared dialogue workshops provided a forum during which expert drawn from the Praslin and Mahe fishers association provided input on local indicators to be considered for the vulnerability assessment.

### Livelihood vulnerability index

Chambers and Conway [49] views on the Sustainable Livelihoods Approach at the household level constitute the following types of assets —natural, social, financial, physical, and human capital. This approach has been used as a guiding framework to design development programmes at the community level [61] despite its limited extent in addresses the issues of sensitivity and adaptive capacity to climate change. However, a pragmatic approach for vulnerability assessment that integrates climate exposures and accounts for household adaptation practices with a comprehensive evaluation of livelihood risks resulting from climate change was developed by a Mozambican study [57]. This study applied the Livelihood Vulnerability Index (LVI) to estimate the differential impacts of climate change on communities in two districts of Mozambique and the same approach is adopted in the current study.

The LVI uses multiple indicators to assess exposure to natural disasters and climate variability, social and economic characteristics of households that affect their adaptive capacity, and current health, food, and water resource characteristics that determine their sensitivity to climate change impacts. In the Seychelles case study, water resources is not considered given that the Public Utilities Corporation (PUC) is the sole provider of water with households assumed to have equal access to water resources and tariffs per unit are the same [62]. A study conducted in the Tigray Region of Ethiopia showed that householders access water from dugout ponds and rivers with intense competition for domestic, livestock and agricultural uses with water shortages during most of the extended drought period as a consequence of climate change [63]. Similar causes of water conflicts amplified by climate variability and change were reported by another study in Zambia [64]. However, the context is different in Seychelles and water is not a suitable major component for fishers when conducting a vulnerability assessment.

Two approaches are presented for calculating the LVI: the first expresses the LVI as a composite index comprised of six major components (socio-demographic profile [SDP], livelihood strategies [LS], food [F], health [H], social networks [SN], and natural disasters and climate variability [NDCV]), while the second aggregates these six components into IPCC’s three contributing factors to vulnerability—exposure, sensitivity, and adaptive capacity. By using primary household data among small-scale fishers, this approach helps avoid the pitfalls associated with using secondary data. Another advantage is the reduction in dependence on climate models, which despite recent advances are still presented at too large a scale to provide accurate projections at levels useful for community development planning [65].

## Method

### Calculating the LVI: composite index approach

Guided by an earlier study on LVI conducted in Mozambique [57] in addition to literature review on the topic of vulnerability, the following six major components were selected: SDP, LS, F, H, SN and NDCV. Each of the major component consists of sub-components (Table 1). These major and sub-components were developed based on a review of literature, for example studies on the impacts of climate change on the fishery sector in Seychelles, as well as expert judgement (representatives from fishers association on Mahe and Praslin Island) and the practicality of collecting the required data through household surveys among small-scale fishers. An explanation on the data that was collected for each component to address vulnerability is presented in Table 2.

**Table 1:** Indicators used to determine fishery-based livelihood vulnerability

Indicators	Explanation of the indicators	Sources of data
Indicators of exposure		
See Table 2 Indicators of Sensitivity	See Table 2	See Table 2
Employment from fisheries	Number of days a household is involved with fisheries in last year	Household questionnaires (HQs)
Income from fisheries	Percentage of household income from fisheries sector in last year	HQs
Nutrients uptake from fisheries	Amount (per capita) of fish and seafood a household consumed in last year (kg/month)	HQs
Indicators of Adaptive Capacity		HQs
Adult workforce	Number of individuals aged 14–60 in household	HQs
Presence of non-elderly household head	Whether household head is\50 years old or not	HQs
Experience	Experience of household head in fisheries-related activities (number of fishing years)	HQs
Education	Number of schooling years of household head	HQs
Livelihood diversification	Average livelihood diversification index calculated as 1/# livelihoods.	HQs
Health-related	Number of days a year household head remains physically fit to carry out livelihood activities.	HQs
Natural capital	Aggregate index of natural capital based on possession of land with index ranging between 0 and 1.	HQs and Interviews
Financial capital excluding income	Aggregate index of household financial capital excluding income and calculated as sum of household scores (no = 0, yes = 1) based on 4 variables; (i) owner of boat, (ii) accommodation, (iii) car, and (iv) television.	HQs

Despite the major components having varied numbers of sub-components, a balanced weighted average approach is applied in the LVI where each sub-component contributes equally to the overall index [66]. Given that each of the sub-components is measured on a different scale, it is imperative to standardize each as an index. Therefore, the approach used in the Human Development Index to calculate the Life Expectancy Index, which is the ratio of the difference of the actual life expectancy and a pre-selected minimum, and the range of pre-determined maximum and minimum was adopted [67] as follows:

1 $${index}_{si}=\frac{{S}_{i}-{S}_{min}}{{S}_{max}-{S}_{min}}$$

where index *S*_*i*_ is the original sub-component for island I, and *s*_*min*_ and *s*_*max*_ are the minimum and maximum values, respectively for each sub-component determined using data from both islands. These minimum and maximum values were used to transform the indicators into a standardized index for ease of integration into the corresponding major components. For example, variables that measured frequencies such as the ‘percent of household income from fisheries sector during the last year’, had the minimum value set at “0” and the maximum at “100”. Some sub-components such as the ‘Average Livelihood Diversification Index’ were created because an increase in the crude indicator, in this case, the number of livelihood activities undertaken by a household, was assumed to decrease vulnerability. In other words, the assumption was that small-scale fishers who owner transportation business, a guesthouse, or engage in carpentry works are less vulnerable that those dependent solely on fishing. By taking the inverse of the crude indicator, a number was created that assigns higher values to households with lower number of livelihood activities. The maximum and minimum values were also transformed following this logic and Eq. (1) used to standardize these sub-components.

The next step after the standardization process was to average the sub-components in order to calculate the value of each major component using Eq. (2) below:

2 $${M}_{i}=\frac{{\sum}_{i=1}^{n} index {s}_{{d}^{i}}}{n}$$

where M_i_ = one of the six major components for island I (SDP, LS, F, H, SN and NDCV), ${index}_{{s}_{{d}^{i}}}$ represents the sub-component, and *n* is the number of sub-components in each major component. After the values for each of the six major components for an island were calculated, they were averaged to obtain the island-level of LVI among fishing households using Eq. (3):

3 $${LVI}_{i}=\frac{{\sum}_{i=1}^{6} {W}_{{M}_{i}} {M}_{{d}_{i}}}{{\sum}_{i=1}^{6} {W}_{{M}_{i}}}$$

Equation 3 can also be expressed as follows:

4 $${LVI}_{i}= \frac{{w}_{SDP}{SDP}_{i}+{w}_{LS}{LS}_{i}+{w}_{F}{F}_{i}+{w}_{H}{H}_{i}+{w}_{SN}{SN}_{i}+{w}_{NDCV}{NDCV}_{i}}{{w}_{SDP}+{w}_{LS}+{w}_{F}+{w}_{H}+{w}_{SN}+{w}_{NDCV}}$$

where${LVI}_{i}$, is the LVI for island *I*, equals the weighted average of the six major components. The weight of each major component, ${w}_{Mi}$ are determined by the number of the corresponding sub-components that make up the major component and are included to ensure that all sub-components contribute equally to the overall LVI [66]. The LVI is scaled from 0 (least vulnerable) to 0.5 (most vulnerable). A detailed step-by-step procedure on how the LVI has been calculated for all six major components and their corresponding sub-components for fishing households on Mahe and Praslin are presented in Appendix A1–A6.

### Calculating the LVI-IPCC: IPCC framework approach

An alternative method for calculating the LVI that takes into consideration the IPCC vulnerability definition was developed under the following categories-exposure, sensitivity and adaptive capacity (see Table 1). Exposure of the study population (small-scale fishing households on Mahe and Praslin) is measured by the number of natural disasters that have occurred in the past 10 years, while climate variability adopting a similar method by Islam et al. 2013 [32] is measured by the average standard deviation of the maximum and minimum monthly temperatures and monthly precipitation between 1993 and 2020 (see Table 2).

As illustrated in Table 1, the adaptive capacity is quantified by the socio-demographic profile of small-scale fishing households on Mahe and Praslin (e.g. percent of female-headed households), the type of livelihood strategies employed to ensure diversification (e.g. predominantly fishing, or also engages in other income generating activities), and the strength of social networks (e.g. percent of fishers that receives assistance in times of crisis). Lastly, sensitivity is measured by assessing the current state of fishing households on both islands in terms of food and health status. The sub-components outlined in Tables 1 and 2 as well as Eqs. (1–3) were used to calculate the LVI-IPCC. It should be noted that, the LVI-IPCC diverges from the LVI when the major components are combined. Therefore, rather than to merge the major components into the LVI in one step, they are first combined into the categorization scheme in Table 3 using the following equation:

**Table 2:** Community exposure to climatic shocks and stresses

Climatic shocks and stresses	Mahé		Praslin		Sources of data
	Mean	Standard deviation	Mean	Standard deviation	
Number of past floods	1.67	0.47	1.75	0.83	Seychelles National Integrated Emergency Management plan^a^/Interviews
Number of past landslide	2	1.22	3.33	1.25	Seychelles National Integrated Emergency Management plan^a^/Interviews
Number of past storm	1.5	0	1	0	Seychelles National Integrated Emergency Management plan^a^/Interviews
Past land erosion (m/year)	0.23	0.09	0.21	0.07	Coastal Management Plan^b^
Maximum temperature (°C)	30.06	0.35	30.09	0.38	Seychelles Meteorological Authority (SMA)
Minimum temperature (°C)	26.08	0.58	26.30	0.58	Seychelles Meteorological Authority (SMA)
Past rainfall (mm)	2335.95	387.95	1994.30	428.68	Seychelles Meteorological Authority (SMA)

^a^ World Bank and DRDM [68]

^b^ World Bank and MEECC [69]

5 $${CF}_{i}=\frac{{\sum }_{i=1}^{n}{w}_{{m}_{i}} {m}_{{d}_{i}}}{{\sum }_{i=1}^{n}{w}_{{m}_{i}}}$$

where ${CF}_{i}$ is an IPCC-define contributing factor (exposure, sensitivity, or adaptive capacity) for island *i*, ${M}_{di}$ are the major components for island *i* indexed by *i*, ${w}_{Mi}$ is the weight of each major component, and *n* is the number of major components for each contributing factor. After the calculation of exposure, sensitivity, and adaptive capacity, these three contributing factors that determine the level of vulnerability were combined using the following equation:

$$LVI-{IPCC}_{i}=\left({e}_{i}-{a}_{i}\right)*{s}_{i}$$

where LVI-IPCC_*i*_ is the LVI for island *i* expressed using the IPCC vulnerability framework, *e* is the calculated exposure score for island *i* (equivalent to the NDCV major component), *a* is the calculated adaptive capacity score for island *i* (weighted average of the SDP, LS and SN components) and *s* is the calculated sensitivity score for island *i* (weighted average of the H and F major components). The LVI-IPCC is scaled from -1 (least vulnerable) to 1 (most vulnerable).

**Table 3:** Categorization of major components into contributing factors from IPCC vulnerability definition for calculation of the LVI-IPCC

IPCC contributing factors to vulnerability	Major components
Exposure	Natural disasters and climate variability (NDCV)
Adaptive capacity	Socio-demographic profiles (SDP) Livelihood strategies (LS) Social networks (SN)
Sensitivity	Food (F) Health (H)

### Study area

The Seychelles is an archipelago of 115 islands in the Indian Ocean, off the coast of East Africa with an estimated population of 98,462 as of 2020 and a land area of 455 km^2^ [70]. Mahe and Praslin Islands are the case study locations with a land surface area and population of 157.3 km^2^ and 86,195 for the former and 38 km^2^ and 8737 for the latter. Major livelihood activities in the Seychelles are climate sensitive and include fisheries, agriculture, and tourism that are directly impacted by climate change while other sectors are affected indirectly by same stressor [71]. The sea surface temperature (SST) in Seychelles waters is characterized by two maxima and minima which are linked to the transition period associated with the monsoon and Indian Ocean Equatorial current [72]. The primary minima occur in July–August when the Southeast Monsoon is at its peak and the sun is in the northern hemisphere. Between the months of July–August, the SSTs may be as low as 24.0 °C. On the other hand, the secondary maxima occur between November and February which is immediately followed by the secondary minima while the difference between the secondary maxima and minimum is estimated at 1 °C. Overall, Seychelles has a mean SST of 27.96 °C with a standard deviation of 1.5 °C [72].

Both islands are exposed to sea level rise, temperature and rainfall variation, coastal erosion and the impacts of the southeast monsoon [69, 73], although Praslin which is four times small than Mahe in land area is expected to be more vulnerable to coastal erosion and sea-level rise. The number of small-scale fishers has increased during the last two decades and now accounts for approximately 14% of all households. This increase is also reflected in the number of householders that own a fishing gear, those that work as part of a fishing crew, fish processors and even the sale of fish. However, the fishery sector is exposed to climate-induced vulnerabilities such as changes in wind pattern and increased risk due to relatively stronger winds during the southeast monsoon and also the loss of corals caused by ocean warming with negative impacts on the economy and local livelihood [74].

The fisheries sector in Seychelles waters consists of three main segments:

i. the small-scale fleet, which uses small motorised vessels that fish for demersal and semi-pelagic species in the local area;
ii. the semi-industrial fleet, which uses longliners that are between 14 and 22 m in length and that catch large pelagic species (mainly tuna and swordfish); and.
iii. the industrial fleet, which uses large purse seiners and longliners that are generally foreign owned and concentrate on fishing for tunas (skipjack tuna, yellowfin tuna and bigeye tuna).

There is a large cannery (operated by Indian Ocean Tuna), which has a capacity of 350 tonnes of tuna per day and which mainly supplies the export market. The Indian Ocean Tuna Ltd (IOT) factory was opened in 1987 in the fishing port of Victoria. The IOT has the only frozen product storage facilities in the Seychelles, the capacity of which allows for 25 days of production and also has a fishmeal production factory, which uses the cannery’s by-products and certain by-catches of the fleet. The port of Victoria is managed by the Seychelles Port Authority (SPA) and has a commercial port and a fishing port, which in turn has one part for purse seiners and another part for longliners. At the port there are also businesses that provide services and supplies to the vessels. However, there are no businesses offering frozen product storage services, which is one of the reasons why the Asian longliners do not dock in Victoria. There are six ice plants on Mahé, which produce 35 to 40 tonnes of ice per day. There is also an ice plant on Praslin, which produces 3 tonnes per day. Due to the increased catches and operational peaks of the semi-industrial longliners, there are repeated shortages of ice, which the Seychelles Fishing Authority (SFA) is trying to solve [72].

The vast majority of fishes found in Seychelles are wide ranging species that extend across the Indian Ocean to the western or mid Pacific Ocean. A total of 1196 marine species belonging to 140 families have been recorded in the Seychelles [75]. However, a relatively low percentage of these species are targeted by any sort of fishery. For example, the major fish species targeted by small-scale fishers include Trevally, Red snapper, Jobfish, Emperors, Groupers, Rabbitfish, Mackerel, and other Pelagics. The semi-industrial fishery is comprised of monofilament longline and the major fish species targeted are Swordfish, Yellowfin tuna, Big eye tuna, and other species of Sailfish, Marlin and Sharks. Furthermore, the industrial fishing sector in Seychelles is made up of foreign owned purse seiners and industrial waters longliners operating under license agreement within Seychelles’ Exclusive Economic Zone (EEZ). Main fish species targeted include Skipjack, Yellowfin tuna, Big eye tuna, Albacore, and other Pelagic species [76]. The two most important companies in the marketing of the Seychelles small-scale fleet catch are Oceana Fisheries Co. Ltd and Sea Harvest. Both companies are linked to two others that own semi-industrial longline fleets, which essentially fish for Swordfish. The small-scale fleet products that are sold are the southern red snapper (*Lutjanus purpureas*) and, to a lesser extent, carangids, various other snappers, groupers, threadfins and octopus. There are four recognised export companies: Indian Ocean Tuna (IOT), Oceana Fisheries, Sea Harvest and Ocean Product Seychelles (OPS). Their main markets are France, Germany, Japan, Mauritius, Réunion and the United Kingdom. The semi-industrial longline fleet catch (60% swordfish and 40% tuna) is generally exported in refrigerated transport to the European Union (France, Italy and the United Kingdom) and, to a lesser extent, to Japan [72].

The artisanal fishery in Seychelles comprises all domestic fisheries, including boats ranging in length from 4 to 15 m and the main gears in this sector include hook and line, drop-lines, traps and nets that are used to catch a diverse array of demersal and pelagic fish species [76]. Furthermore, the gear or vessel type in terms of size, engine capacity, available technology also have an impact on the length of fishing trip and fishing range (distance covered) of fishers. Artisanal fishing practices on Mahe are similar to those on Praslin including the type of fishing gears used and the number of days spent in the sea. The major difference is that Mahe is more developed in terms of infrastructure and all the large fish processing plants that export fish abroad are around the Port of Victoria. Additionally, Praslin has stronger presence of community life with just a fishers association which can speak with one voice of issues that affect their professor whereas on Mahe island, there are over five different fishers association and coordinating these groups has proven to be challenging. Approximately 470 vessels are currently active in the artisanal sector, whereby 75% of the fleet are outboard vessels and 20% are whalers. The principal gears used by whalers and schooners are handlines, while the small boats uses a multitude of gear combinations, including handlines, traps, encircling gill nets, beach seines and harpoons [77].

### Household surveys

Information from Table 1 was used to design the household questionnaire based on the six major components for this study to assess the vulnerability of fishing households. Three shared dialogue workshops (SDW) were conducted with representatives from four fishers association (3 from Mahe and the only association on Praslin). A SDW is a forum that brings academics as well as community leaders and household members together at regular intervals to discuss issues of concern patterning to the management of a natural resource [78] and in this case—small-scale fishers and how they are affected by climate and non-climatic drivers of change. During these SDWs, questions were asked regarding climate variability and change within the ocean space (e.g. wind speed, wind direction, roughness of the sea, impacts of the monsoon seasons, coral bleaching events, among others) and how they affect small-scale fishers. Historical data was also gathered during the SDWs on changes that have occurred regarding fishing grounds, drivers of change and how it affects local livelihoods. Lastly, these workshop also provided an opportunity for fishers to discuss the type of incentives they receive from the government (for both association and non-association members) and also livelihood diversification opportunities for fishing households on Mahe and Praslin.

While secondary data were used for the analysis of exposure (see Table 2), a survey was conducted among 80 randomly selected fishing households (40 households in each island) to provide data for sensitivity and adaptive capacity. These surveys were conducted face-to-face and also via the phone given that mid-way into the research was the enforcement of COVID-19 restrictions to movement. However, gathering of less than five persons were still possible with the use of face mask and the respect of physical distances. Telephone numbers were provided by the secretaries of fishers’ association e.g. Fishing Boat Owners Association (FBOA), Glacis and Port Glaud Fishers Associations on Mahe, and the Praslin fishers association. Household head and fishers that participate in the study gave verbal consent after haven received a brief explanation on what the research is about and the type of information that will be needed to complete the survey. The household survey consisted of six sections: SDP, LS, F, H, SN, and NDCV. The completion time of a survey lasted for 30 to 40 min. Interview were conducted in Creole and English depending on the language the household head was comfortable in using. The interviewers re-assured the participants that their names will not be mentioned and the purpose of the survey is purely academic.

**Table 4:** Livelihood Vulnerability Index (*LVI*) sub-component values and minimum and maximum sub-component values for Mahe and Praslin

Major component	Sub-component	Units	Mahe Island	Praslin Island	Maximum value in both islands	Minimum value in both islands
Socio-demographic profile	Dependency ratio	Ratio	0.008	0.007	0.015	0
	% of female-headed households	Percent	0.425	0.325	100	0
	Average age of female head of HH	1/Year	0.022	0.017	0.013	0.037
	% of heads of HH that completed just primary education	Percent	0.075	0.075	100	0
Livelihood strategies	% of HH dependent solely on fisheries as source of income	Percent	95	97	100	0
	% of HH with family member employed outside the fishery sector	Percent	1.625	0.9	100	0
	Average livelihood diversification index	1/# livelihoods	0.38	0.33	1	0.2
Social networks	% of HH that belong to fishers association	Percent	92	98	100	0
	% of HH that belong to other community based organizations	Percent	10	20	100	0
	Percentage of HH that have not gone to the bank for assistance during the past 12 months	Percent	70	60	100	0
Health	% of HH that remains physically fit during the last 12 months to carry out livelihood activities	Percent	97	93	100	0
	Distance to the nearest health center (Minutes)	Minutes	30	20	60	10
Food	% of HH that depend solely on fish/seafood in the last 12 months	Percent	20	35	100	0
	% of heads of HH that get fish from other fishers during the last 12 months	Percent	15	50	100	0
	Average number of months HH struggle to find fish due to monsoon	Months	3	2	5	1
	Fish diversity index	1/# fish species caught +1	1.14	1.13	1.17	1.08
Natural disasters and climate variability	Average number of floods during (years: 1997–2021)	Count	1.67	1.75	3.42	1
	Average number of landslides (years: 1997-2021)	Count	2	3.33	5.33	1
	Average number of storms (years: 1997–2021)	Count	1.5	1	2.5	1
	Average past land erosion in mm/years (years: 2000–2019)	mm	0.23	0.21	0.44	0.01
	Mean standard deviation of yearly average maximum temperatures (years: 1993–2020)	Celsius	0.58	0.58	0.59	0.36
	Mean standard deviation of yearly average minimum temperatures (years: 1993–2020)	Celsius	0.35	0.38	0.59	0.36
	Mean standard deviation of yearly average precipitation (years: 1993–2020)	Millimetres	2435.4	1994.3	4429.7	1

## Results

The results are based on the 80 completed surveys among fishing households with 40 each for Mahe and Praslin Island. The IPCC contributing factors to vulnerability (as presented in Table 3) that takes into account the six major components serve as a guide to the presentation of results in thematic areas of exposure, sensitivity and adaptive capacity.

### Exposure compared between fishing communities

Both islands had similar Natural Disaster vulnerability scores for some of the sub-components while slight differences were observed for others. The average number of floods during the years 1997 to 2021 on Mahe is 1.67 and 1.75 for Praslin (Table 4). From the years 1997 to 2021 the average number of landslides reported was 2 versus 3.33 on Mahe and Praslin indicating that more landslides occurred on Praslin. During the years 1997 to 2021 the average number of storms reported for Mahe was 1.5 and 1 for Praslin (Table 4). Furthermore, the average recorded erosion between the years 1997 to 2019 was 0.23 mm for Mahe and 0.21 mm for Praslin. Both islands had similar mean standard deviation of yearly average maximum temperatures during the years 1993 to 2020 corresponding to 0.58 degrees Celsius. The mean standard deviation of yearly average minimum temperatures on Mahe was 0.35 degrees Celsius during the years 1993 to 2020 while Praslin recorded 0.38 degrees Celsius during the same period (Table 4). Mahe recorded 2435.4 millimeters of the mean standard deviation of yearly average precipitation during years 1993–2020 whereas the recorded value for Praslin was much lower (1994.3 millimeters). When the seven sub-components were averaged, the overall Climate disaster and climate variability vulnerability score was higher for Praslin (0.4) than on Mahe (0.26).

### Sensitivity compared between fishing communities

The percentage of household dependent solely on fisheries as source of income based on the survey results is 95% on Mahe and 97% for Praslin—an indication that employment along the value chain working as transporters, fish mongers and processors is common among fishing households. The percentage of household with family member employed outside the fishery sector are relatively small corresponding to 2% on Mahe compared to a miniscule 0.9% for Praslin. This further explained the slight difference in terms of livelihood diversification on Mahe (0.38) and 0.33 on Praslin (see Table 5) with the former benefiting from the diverse opportunities associated with its level of development. In this regard, the participants of the SDWs further revealed that fishers also gained income from tourism-related activities such as boat ride for both tourist and the local population, income from the sales of coconuts, carpentry works, car rental services, guest house, among others.

**Table 5:** Indexed sub-components, major components, and overall LVI for Mahe and Praslin Islands, Seychelles

Sub-component	Mahe Island	Praslin Island	Major component	Mahe Island	Praslin Island
Dependency ratio	0.53	− 0.008	*Socio- demographic profile*	**0.29**	**0.21**
% of female-headed households	0.004	0.0325			
Average age of female head of HH	0.625	0.833			
% of heads of HH that completed just primary education	0.0008	0.00075			
% of HH dependent solely on fisheries as source of income	0.95	0.97	*Livelihood strategies*	**0.4**	**0.38**
% of HH with family member employed outside the fishery sector	0.01625	0.009			
Average livelihood diversification index	0.225	0.1625			
% of HH that belong to fishers association	0.92	0.98	*Social networks*	**0.57**	**0.59**
% of HH that belong to other community based organizations	0.1	0.2			
Percentage of HH that have not gone to the bank for assistance during the past 12 months	0.7	0.6			
% of HH that remains physically fit during the last 12 months to carry out livelihood activities	0.97	0.93	*Health*	**0.69**	**0.565**
Distance to the nearest health center (Minutes)	0.4	0.2			
% of HH that depend solely on fish/seafood in the last 12 months	0.2	0.45	*Food*	**0.373**	**0.39**
% of heads of HH that get fish from other fishers during the last 12 months	0.15	0.5			
Average number of months HH struggle to find fish due to monsoon	0.5	0.25			
Fish diversity index	0.64	0.36			
Average number of floods during (years: 1997–2021)	0.28	0.31	*Natural disasters and climate* *Variability*	** 0.26**	** 0.40**
Average number of landslides (years: 1997–2021)	0.23	0.54			
Average number of storms (years: 1997–2021)	0.33	0			
Average past land erosion in mm/years (years: 2000–2019)	0.51	0.47			
Mean standard deviation of yearly average maximum temperatures (years: 1993–2020)	− 0.04	0.96			
Mean standard deviation of yearly average minimum temperatures (years: 1993–2020)	− 0.04	0.09			
Mean standard deviation of yearly average precipitation (years: 1993–2020)	0.55	0.45			
Overall LVI				** 0.381**	**0.402**

The bold values are the vulnerability index for Mahe and Praslin. The higher the value in bold, the more its vulnerability for each of the major component and the lower the value, the lesser the vulnerability.

Physical fitness and time taken to access health facilities were the two components considered for health. A total of 97% of households on Mahe were physically fit during the last 12 months prior to the survey to carry out livelihood activities compared to 93% on Praslin (Table 5). Mahe householders reported traveling an average of 30 min to the nearest health facility whereas those on Praslin travelled an average of 20 min based on private transportation. It was reported during the SDWs that it’s difficult to find a fishing household in the Seychelles that does not own a car and therefore, ease of traffic is a key determinant to travel time. For the combined health sub-components, the overall health vulnerability score for Mahe Island (0.69) is higher than that for Praslin (0.57). Aside from the waiting time to see a doctor which is much longer on Mahe due to its population, traffic congestion is another problem that might increase travel time to a health centre during peak period especially in the morning and lunch period.

The overall food vulnerability score for Mahe is lower 0.373 than that of Praslin 0.39. On Mahe Island, 20% of the householders depend mainly on fish or seafood in the last 12 months compared to 35% on Praslin. It is reported that 15% heads of households on Mahe reported to have received fish from other fishers during the last 12 months whereas 50% of households on Praslin did receive similar support. Fishers hardly preserve fish in refrigerators but prefer to consume those that have just been caught. Some of the reasons that often lead to fisher resorting to borrow fish from colleagues for consumption were roughness of the sea during the monsoon season, health issues, or other engagements. The fish diversity index for both islands were similar (see Tables 4 and 5) indicating that fishers have access to same species. However, the southeast monsoon affects the fishers differently with Mahe householders struggling to find fish for 3 months compared to 2 months for those on Praslin during the monsoon season.

### Adaptive capacity compared between fishing communities

Going by results presented in Table 5, Mahe Island had a dependency ratio index slightly higher (0.008) than Praslin Island (0.007). Overall, small-scale fishers on Mahe showed greater vulnerability for *SDP* index compared to Praslin (*SDP*_*Mahe*_ 0.29; *SDP*_*Praslin*_ 0.21). The percentage of female-headed households was 42.5% for Mahe and 32.5% for Praslin indicating that more female-headed households occurred in the former than the latter. In addition, the average age of the female heads of households demonstrated that younger female heads occurred on Mahe than Praslin Island corresponding to 45 and 57 years respectively. Also, the percentage of heads of household that completed just primary education was the same for both fishing communities and stood at 7% (Table 5). Information gathered during the SDWs indicated that education since the last two decades in state-owned institutions up to post-secondary is highly subsidized by the government. This partly explains why majority of the fishing households were able to attain education beyond the primary level.

The social network indicators were similar for both island and 92% of Mahe and 98% of Praslin households were registered members of a fishers association. Mahe households reported that 10% belong to other community based organizations compared to 20% for those on Praslin. More importantly, 70% and 60% of the fishing households on Mahe and Praslin have not gone to the bank for assistance during the past 12 months. The livelihood strategies for fishing households on both island are not too different from each other corresponding to 0.4 and 0.38 for Mahe and Praslin respectively (Table 5). Overall, Praslin households showed greater vulnerability (0.59) than those on Mahe (0.57) in terms of the social networks component.

### The LVI-IPCC assessment compared between fishing communities

**Fig. 2:**
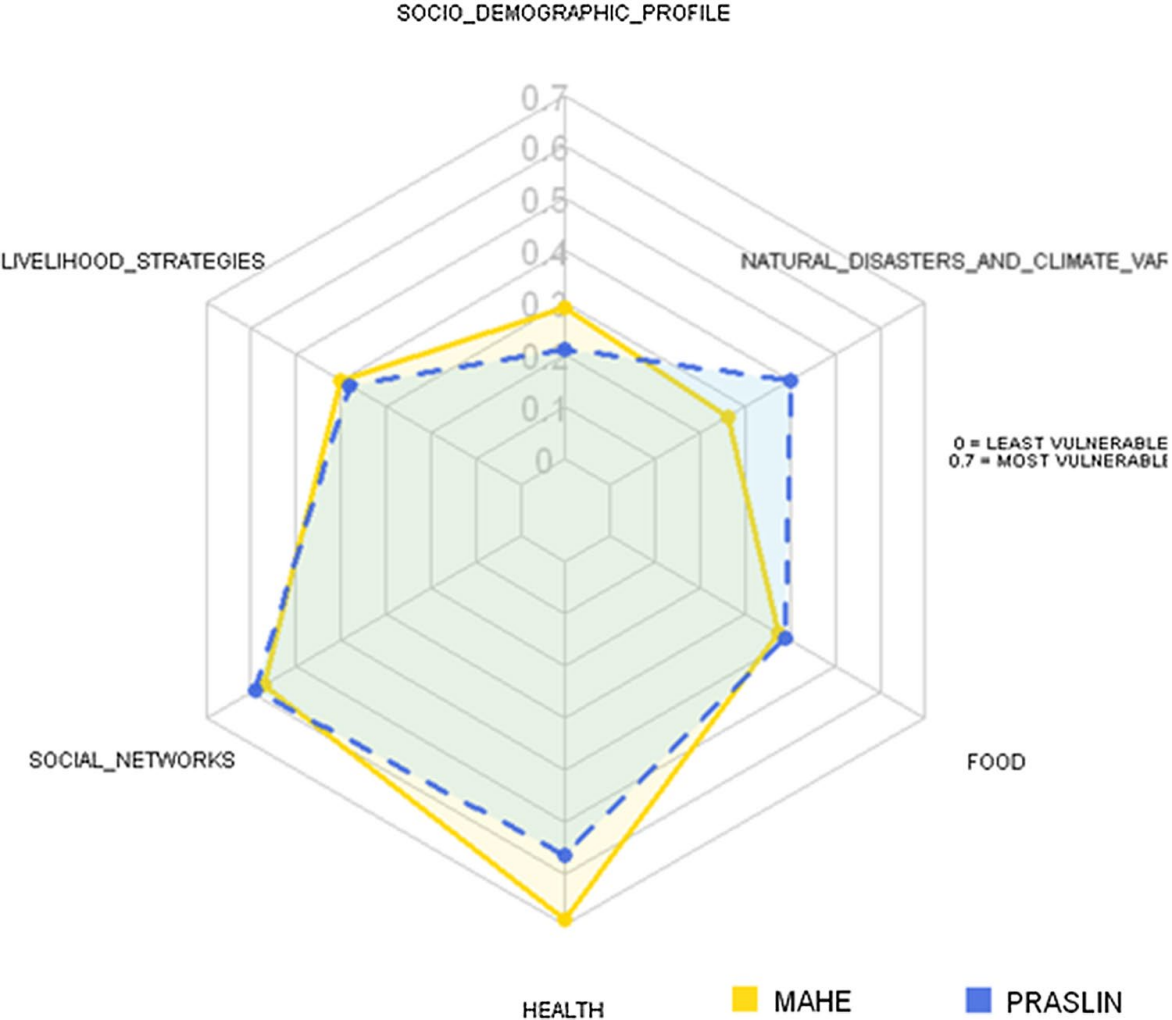
Vulnerability radar chart of the major components of the Livelihood Vulnerability Index (LVI) for Mahe and Praslin Island

Praslin Island had a higher LVI than Mahe 0.381 versus 0.402, indicating relatively greater vulnerability to climate change impacts. The results of the six major components are further presented collectively in a radar chart (Fig. 2). The scale of the diagram ranges from 0 (less vulnerable) at the center of the web, and then increasing to 0.7 (more vulnerable) at the outside edge in 0.1 unit increments. Figure 2 indicate that Mahe Island is more vulnerable in terms of Health (0.69), while Praslin Island is more vulnerable in terms of Social Networks, food, and natural disaster and climate variability.

**Fig. 3:**
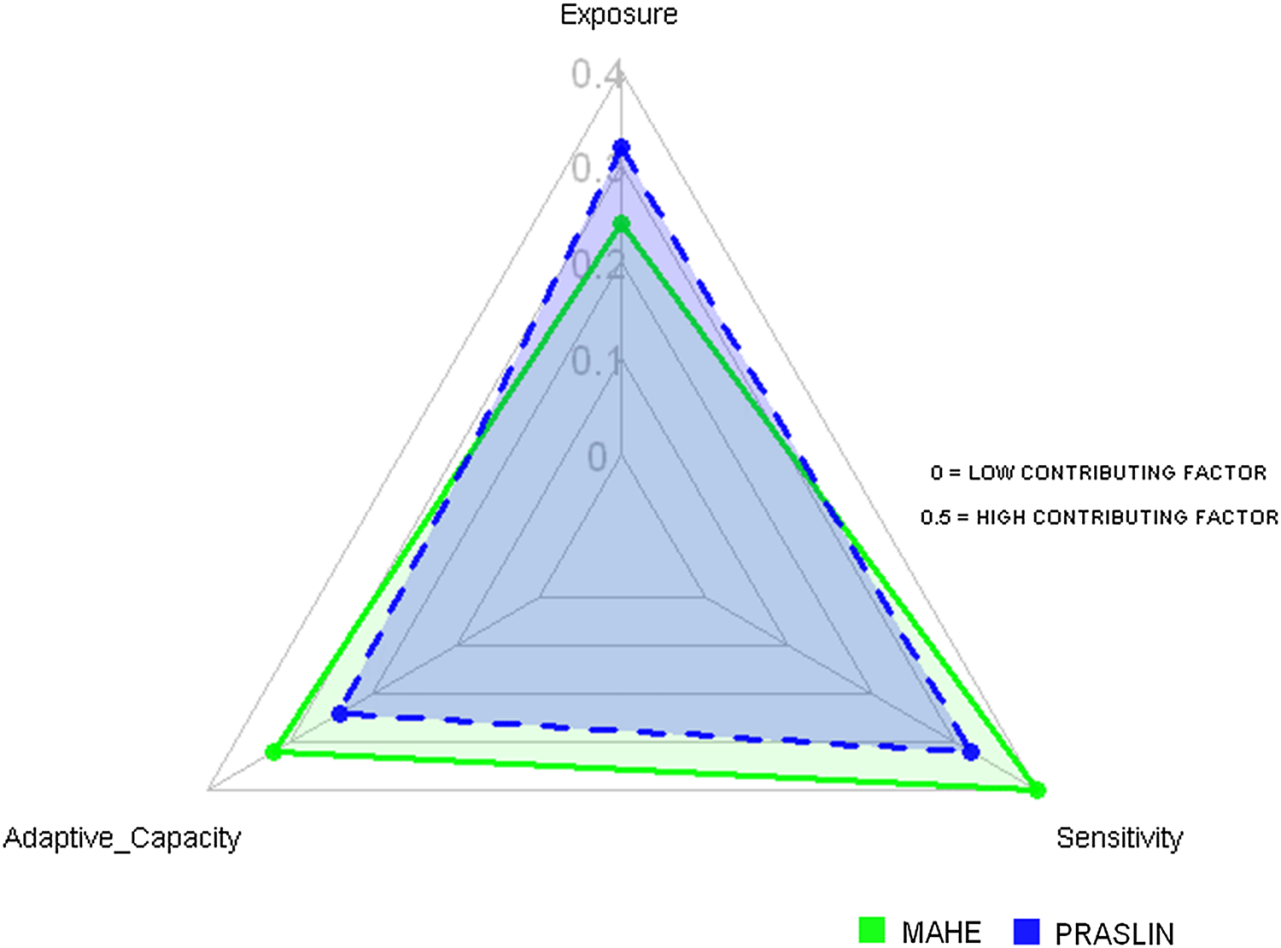
Vulnerability triangle diagram for the contributing factors of the Livelihood Vulnerability Index-IPCC (LVI-IPCC) for Mahe and Praslin Island

The LVI–IPCC analysis as presented in Table 6 yielded similar results (LVI–IPCC: Mahe 0.07, Praslin 0.011). A vulnerability triangle plots that shows the contributing factor scores for exposure, adaptive capacity, and sensitivity are presented in Fig. 3. The triangle explains that Praslin may be more exposed (0.4) to natural disasters and climate variability than Mahe (0.26). Nevertheless, taking into account the health major component, Mahe may be more sensitive than Praslin (0.479 versus 0.448, respectively). Based on socio-demographic profile, livelihood strategies, and health, Mahe showed a lower adaptive capacity 0.407 versus 0.375 for Praslin. The overall LVI–IPCC scores indicate that Praslin households may be more vulnerable than Mahe households (0.011 versus − 0.07, respectively).

**Table 6:** LVI–IPCC contributing factors calculation for Mahe and Praslin Islands, Seychelles [54]

IPCC contributing factors to vulnerability	Mahe Island	Praslin Island
Exposure	0.26	0.4
Adaptive capacity	0.407	0.375
Sensitivity	0.479	0.448
LVI-IPCC	− 0.07	0.011

## Discussion

The "discussion" section just like the results is presented according to the following thematic areas; exposure, sensitivity and adaptive capacity between fishing communities in the two case study island. Based on the LVI-IPCC methodology, an overall vulnerability for both islands are summarized at the end of this section.

### Exposure between fishing communities

Praslin showed greater overall vulnerability when compared to Mahe for *NDCV* which was also the case for the sub-component of floods and landslides. Generally, the number of flooding events, storms and past land erosions have increased in terms of frequency during the last 30 years in the Seychelles as documented during the SDWs—a view support by Etongo et al. [79]. According to the World Bank and MEECC [69], the minimum and maximum past land erosion in mm/year between 2000 and 2019 for Mahe and Praslin was 0.01mm and 0.44mm. The figure was slightly higher for Mahe (0.23 mm/year) when compare to Praslin (0.21 mm/year) indicating that sea level rise will exacerbate the impacts of coastal erosion on coastal communities and infrastructures that support fishing livelihoods—a view supported by several other studies in developing countries [15–19, 12]. Information gathered during SDWs from the fishers showed that the Port of Victoria on Mahe Island experiences high tides of up to 1.9 m above Mean Sea Level while on Praslin the figure is 1.75 m. Although high tides are normal and occur every month in relation to a full moon cycle, its impact in some cases have affected coastal infrastructures that support the fishery sector with most of these infrastructures viewed as inadequate especially among small-scale fishers in developing countries [23–25]. Furthermore, the Seychelles oceanographic climate is dominated by two seasons: southeast monsoon (SEM) which runs roughly from April through September, and northwest monsoon (NEM) which runs roughly from October through March. Both seasons have varied impacts on small-scale fishers in terms of access to fish stock especially during the SEM. For example, strong winds from the southeast causing locally generated wind waves with short periods mixed with long period swell coming from storms in the southern Indian Ocean. On the other hand, periods of strong NW winds cause locally large waves from the NW but it is much smaller than during the SEM season. During the SDWs, it was mentioned that wind direction in the ocean space is fast changing couple with increased sea roughness especially during the SEM season which seems to affect the fishers. The main system that governs weather over this part of the Indian Ocean is primarily the Intertropical Convergence Zone (ITCZ) [80].

Climate change projections for the next twenty years show a warming between + 0.33 °C and + 0.82 °C respectively [81] and global warming has been seen to transform coral reef assemblages in the Great Barrier Reef [15]. Ocean warming leading to coral bleaching is evident in Seychelles with economic and environmental consequences. For example, the mass coral-bleaching event of 1997–1998 is considered the most severe on record [82]. Bleaching was particularly severe in the Indian Ocean, with post-bleaching levels of mortality reaching as much as 90% in parts of eastern Africa and the Maldives and 50–90% over extensive areas of shallow reefs in the Seychelles, Comoros, Madagascar and Chagos [83]. The Seychelles suffered another coral bleaching event in 2016 and the resilience of coral is much better in areas with minimal human activities [84]. Aside from ocean warming and its effect on the fishery sector, climate change manifesting through global warming thereby resulting in sea level rise [85, 86]. The two major causes of a global rise in sea level are thermal expansion of the oceans (water expands as it warms) and the increased melting of glaciers and sea-ice. Sea-level rise is of high concern and importance for coastal systems as it could lead to storm surges, coastal flooding, coastal erosion and salinization [87]. Furthermore, there is a high certainty that the sea level will rise to some extent in 95% of the oceans by the end of the century.

### Sensitivity between fishing communities

Results indicated that fishers on Mahe struggled more than those on Praslin for a good catch during the SEM season when the sea shows more roughness. Maintaining fish stock at specific site seems to have worked well for fishers on Praslin compared to those on Mahe. The voluntary closure of some fishing zones has been applied on Praslin—an initiative of the Praslin Fishing Association [88]. This initiative was driven by sustainability of the livelihoods among the small-scale fishers as reported by the chairperson of the Praslin Fishers Association. “*We as fishermen have noticed that the size of fish is decreasing and so is the amount. We have taken examples of similar projects that have been carried out in other places and have had interesting results*”. In order to protect the closure zones, three yellow buoys labelled ‘Fisheries Closure’ were used as indicators across these zones and all vessels are to keep a safe distance and to navigate with extreme precaution when approaching the demarcated areas [89]. The closure period was between the months of November 2019 to April 2020. By the end of the closure period, two main targeted species by small-scale fishers (rabbitfish and parrotfish) both increased by six centimetres [90]. An increase in the size and number of fish caught in traps was observed around the main harbour of Praslin—a strategy that ensures sustainability of fish stock while reducing the vulnerability of fishers especially during the SEM season as closure zones are often utilized during such periods. Praslin householders reported struggling to find fish for an estimated 2 months compared to 3 months for those on Mahe. Therefore, such management action on Praslin hinges on good governance which is one of the core areas promoted through the Code of Conduct for Responsible Fisheries for close to three decades [8, 91].

When combining the two Health sub-components, Mahe Island (0.69) has a greater Health vulnerability score than Praslin Island (0.565). Mahe householders reported a longer average time to reach a health centre, while Praslin has a shorter time, due to the fact that Praslin Island is smaller than Mahe Island and has less vehicle, hence less traffic. However, the percentage of Mahe households that remain physically fit during the last 12 months to carry out livelihood activities is slightly higher when compared to those on Praslin. That notwithstanding, the Seychelles government provides free access to primary healthcare for all its citizens and has adequate services in child and maternity healthcare. Access to education and health, safe drinking water and sanitation is considered to be adequate within the country. Therefore, vulnerability in terms of health can be better interpreted in terms of travel time to the nearest health facility and also waiting time to see a doctor.

### Adaptive capacity between fishing communities

Fishers on both islands enjoy a range of benefits in the form of subsidies given to small-scale fisheries such as reduced licence fee, business tax exemption, income tax concession, fuel subsidy, ice concession, among others [92]. Despite these range of benefit, subsidies in Seychelles have become one of the most controversial subject more than any other single factor and is viewed as a source to problems such as overfishing, illegal fishing and unfair benefit sharing [93]. It’s important to note that there are about 60 registered boats participating in the small-scale fishery on Praslin, which together provides employment for about 150 fishers. Two thirds of these boats are small out-board engine powered boats locally known as Mini-Mahé, which usually operates within 10 miles from the island. These small boats usually does a mix of trap and hand-line fishing. During the North West Monsoon when the sea is calm, fishers venture further out to fish on the deeper offshore fishing banks where the catch is more abundant. Nevertheless, they also continue to fish extensively on the surrounding fringing reefs because of easy picking. However, the situation is different during the southeast trade winds, when the sea is rough and the number of days that fishers can go beyond the reef is limited especially for those with small boats and most fishing is concentrated in the lagoons. Given that some of the small-scale fishers have acquired long liners, their access to fishery stock even during the southeast monsoon season is relatively much better. This further explains that vulnerability could be spatial and temporal. For example, fishers with small boat may concentrate fishing around the reef or lagoons during the SEM season when the sea is rough. This is the same period that closure zones are implemented on Praslin Island in order to allow for increase in fish size and number, but householders that cannot venture further into the sea are likely to have access to lesser fish stocks. However, it is worth mentioning that fishers on Praslin are allowed to fish in the Baie Ste Anne lagoon during the southeast monsoon in order to augment their income and livelihood given that small-scale fisheries is important for food security and employment especially in developing countries [8–10]. Otherwise the lagoon is closed during the northwest monsoon to allow fish to increase in size and number—an indication group cohesion has an impact on management strategy of fisheries as demonstrated in the case of Praslin when compared to Mahe with several fishers association.

Information gathered from resource persons during the SDWs indicated that small-scale fishers also engage in other income generating activities especially when sea roughness does not allow their engagement in fishing. Examples of other income sources include tourism-related activities, agriculture, carpentry works and transportation services. In the case of Brazil and South Africa, small-scale fishers have historically practiced alongside fishing, small-scale farming, hunting, and extraction of plan resources in the forest [9]. Praslin householders had a higher dependency on solely fish/seafood for the past 12 months, when the fishers are unable to fish they are receiving fish from other fishers. This is due to the fact that community life on Praslin is better developed with lesser population and multiculturalism when compared to Mahe island that demonstrate the opposite given that its host about 90% of the nation’s population. That notwithstanding, the fish diversity index were similar for both island indicating that fishers had access to similar fish species that are supported by the domestic market.

Fishing households on Mahe reported diversifying their income sources beyond fishery by being employed in other sectors such as retail businesses, agriculture, transportation, and in the tourism sector for those that own guesthouse, provide services as tour guide and more importantly boat ride for tourists and even the local population. A small percentage of Mahe householders depended solely on fisheries as a source of income and had a greater average livelihood diversification index. Mahe fishing households are engaged in more than 3 fishery-based activities aside from fishing, to transportation of fish in vans, sale of fish and also provide labour for the processing of fish meant for export, whereas Praslin households had on average two livelihood activities besides fishing—transportation of fish and sale of fish. Mahe being the main island that also host the capital Victoria has more infrastructural development in the fishery sector such as ice plant, processing industries and the international airport to facilitate exportation. Hence Mahe households have a greater adaptive capacity as fishers often engage in the different livelihood opportunities that it present and thus a relatively higher diversification index.

The percentage of household that belong to fishers association is greater on Praslin than on Mahe. Praslin Island being smaller than Mahe Island has more of community spirit rather than Mahe being more business oriented and diversified with several fishers association. This is also reflected by the greater percentage of Praslin households that belong to other community-based organizations. However, Praslin householders recorded a relatively higher percentage that sort financial assistance from the bank during the past 12 months. Praslin Island also suffered greatly from the COVID-19 pandemic that severely impacted the tourism sector which represented a key source of income for diversification. However, COVID-19 relief employment benefits were made available to all registered fishers during part of the year 2020, thereby reducing their vulnerability economically during this period [93].

Overall Mahe Island showed greater vulnerability on the Socio-Demographic Profile. The percentage of female-headed households is higher on Mahe Island 0.425 than Praslin Island 0.325. The reported average age of Mahe female household heads was 2.2% and for Praslin 1.7% while the percentage of heads of household that completed just primary education was similar for both islands and stood at 7.5%. Historically, there has not been any cultural role that imposes restrictions as to who acquire education or perform domestic duties based on gender [94]. Such a deviation from the situation that still prevail in some developing countries have led to varied opinions in the Seychellois society along gender lines as a matrifocal vis-à-vis a matriarchy society. Mothers tend to be dominant in the household, controlling most current expenditures and looking after the interests of the children. Men are important for their earning ability with men earning wages that are 9% higher than women at the 10th percentile and 19% higher at the 90th percentile [95], but their domestic role is relatively peripheral. It is important to consider not just age and sex disaggregated data, but also who has decision-making power at the level of the household in order to avoid misrepresentation of information.

### The LVI–IPCC assessment between fishing communities

Praslin may be more exposed (0.4) to natural disasters and climate variability than Mahe (0.26). Nevertheless, taking into account the health major component, Mahe may be more sensitive than Praslin (0.479 versus 0.448, respectively). Based on socio-demographic profile, livelihood strategies, and health, Mahe showed a lower adaptive capacity 0.407 versus 0.375 for Praslin. The overall LVI-IPCC scores indicated that Praslin householders may be more vulnerable than those on Mahe island (0.011 versus − 0.07, respectively). That notwithstanding, the “fisheries closure” which is a management strategy put in place by the fishers on Praslin provided an opportunity for an increase in size and number of two main targeted species by small-scale fishers.

## Conclusions

While most of the indicators were similar, the overall climate disaster and climate variability vulnerability score was relatively higher for Praslin when compared to the fishing community on Mahe Island. However, fishers on Mahe struggled more to access fish in the ocean especially during the southeast monsoon season while their fellow fishers on Praslin had alternative strategies as a mitigation strategy. For example, fishers on Praslin were allowed access to fish around the lagoon during SE monsoon. Additionally, they had closure zones at specific which resulted to increase in size and number of two important fish species after seven months of closure—a sustainability strategy that has the potential improve the adaptive capacity of small-scale fishers especially under a fast changing climate. Although fishers on both islands had other sources of income within and outside the fishery sector, those on Mahe had greater opportunity for diversification given the level of development and population size of the main island. The level of development also acted as a disincentive when accessing health care services with traffic congestion increasing the time especially during peak period to arrive at a health centre while the relatively higher population implies doctor to patient ratios were higher with an implication on waiting time to see a doctor. The several subsidies provided by the government of Seychelles to the small-scale fishers (e.g. ice and fuel concession, financial support during period of lockdown due to COVID-19) are measures that can go a long way to improve the adaptive capacity of fishers.

One of the novelty of this study is the design of locally sensitive indicators which are important for informing effective interventions especially at the community level. This methodology based on locally design indicators of vulnerability to climate change can be replicated in other fishing communities with similar circumstances. The vulnerability of fishery-based livelihoods on both islands can partly be explained by the level of investment in this sector and the governance structure in place. Infrastructural development such as ice processing plant, landing sites, fish processing plants and access to a much better market is a common feature on Mahe from which the fishers benefit from as income opportunities become available along the value chain. However, the distance travelled by fishers in order to ensure a much better catch has increased over the year partly due to non-climatic drivers such as higher demand for fish and also an increase in the number of small-scale fishers. Therefore, vulnerability of fishery-based livelihoods goes beyond climate drivers to include aspects of investment on infrastructure such as landing sites, appropriate storage facilities and a ready market. In the case of Seychelles, further studies are needed in two key areas: (i) the role of subsidies and sustainable fisheries management, and (ii) a value-chain approach to vulnerability of small-scale fishers within the fishery sector in Seychelles.

## Data Availability

The datasets generated during and/or analyzed during this study are not publicly available due to request of some respondents not to share their data publicly without reasonable permission. Hence, data will be available from the corresponding author on reasonable request and respondents’ permission.

## Appendix

### Appendix A1. Calculating the Natural disasters and climate variability major component

#### Mahe Island, Seychell/es

**Table:**

Sub-components for Natural disasters and climate variability major component	Sub-component values for Mahe Island	Max sub-component value for study population	Min sub-component value for study population	Index value for Mahe Island	Natural disasters and climate variability major component values for Mahe island
Average number of floods during (years: 1997–2021)	1.67	3.42	1	0.28	0.26
Average number of landslides (years: 1997–2021)	2	5.33	1	0.23	
Average number of storms (years: 1997–2021)	1.5	2.5	1	0.33	
Average past land erosion in mm/years (years: 2000–2019)	0.23	0.44	0.01	0.51	
Mean standard deviation of yearly average maximum temperatures (years: 1993–2020)	0.58	0.59	0.36	− 0.04	
Mean standard deviation of yearly average minimum temperatures (years: 1993–2020)	0.35	0.59	0.36	− 0.04	
Mean standard deviation of yearly average precipitation (years: 1993–2020)	2435.4	4429.7	1	0.55	

Step 1 (repeat for all sub-component indicators): $\mathrm{NDCV }1\mathrm{ Mahe island}=\frac{ 1.67-1}{3.42-1}=\frac{0.67}{2.42}=0.28$

Step 2 (repeat for all major components):$\mathrm{NDCV }1\mathrm{ Mahe island}:=\frac{{\sum}_{i=1}^{n} index {s}_{{d}^{i}}}{n}=\frac{0.28+0.23+0.33+0.51\pm 0.04\pm 0.04+0.55}{7}=0.26$

Step 3 (repeat for all study areas): $\mathrm{LVIMahe }= {LVI}_{i}=\frac{{\sum}_{i=1}^{6} {W}_{{M}_{i}} {M}_{{d}_{i}}}{{\sum}_{i=1}^{6} {W}_{{M}_{i}}}=\frac{\left(7\right)\left(0.26\right)+\left(4\right)\left(0.29\right)+\left(3\right)\left(0.4\right)+\left(3\right)\left(0.57\right)+\left(2\right)\left(0.69\right)+(4)(0.373)}{7+4+3+3+2+4}=\frac{8.762}{23}=0.381$

$${\mathrm{LVIPraslin }=LVI}_{i}=\frac{{\sum}_{i=1}^{6} {W}_{{M}_{i}} {M}_{{d}_{i}}}{{\sum}_{i=1}^{6} {W}_{{M}_{i}}}=\frac{\left(7\right)\left(0.4\right)+\left(4\right)\left(0.21\right)+\left(3\right)\left(0.38\right)+\left(3\right)\left(0.59\right)+\left(2\right)\left(0.565\right)+(4)(0.39)}{7+4+3+3+2+4}=\frac{9.24}{23}=0.402$$

#### Praslin Island

**Table:**

Sub-components for Natural disasters and climate variability major component	Sub-component values for Praslin Island	Max sub-component value for study population	Min sub-component value for study population	Index value for Praslin Island	Natural disasters and climate variability major component values for Praslin Island
Average number of floods during (years: 1997–2021)	1.75	3.42	1	0.31	0.40
Average number of landslides (years: 1997–2021)	3.33	5.33	1	0.54	
Average number of storms (years: 1997–2021)	1	2.5	1	0	
Average past land erosion in mm/years (years: 2000–2019)	0.21	0.44	0.01	0.47	
Mean standard deviation of yearly average maximum temperatures (years: 1993–2020)	0.58	0.59	0.36	0.96	
Mean standard deviation of yearly average minimum temperatures (years: 1993–2020)	0.38	0.59	0.36	0.09	
Mean standard deviation of yearly average precipitation (years: 1993–2020)	1994.3	4429.7	1	0.45	

### Appendix A2. Calculating the Socio-demographic profile major component

#### Mahe Island

**Table:**

Sub-components for Socio-demographic profile major component	Sub-component values for Mahe Island	Max sub-component value for study population	Min sub-component value for study population	Index value for Mahe Island	Socio-demographic profile major component values for Mahe Island
Dependency ratio	0.008	0.015	0	0.53	0.29
% of female-headed households	0.425	100	0	0.004	
Average age of female head of HH	0.022	0.013	0.037	0.625	
% of heads of HH that completed just primary education	0.075	100	0	0.0008	

#### Praslin Island

**Table:**

Sub-components for Socio-demographic profile major component	Sub-component values for Praslin Island	Max sub-component value for study population	Min sub-component value for study population	Index value for Praslin Island	Socio-demographic profile major component values for Praslin Island
Dependency ratio	0.007	0.015	0	− 0.008	0.21
% of female-headed households	0.325	100	0	0.0325	
Average age of female head of HH	0.017	0.013	0.037	0.833	
% of heads of HH that completed just primary education	0.075	100	0	0.00075	

### Appendix A3. Calculating the Livelihood strategies major component

#### Mahe Island

**Table:**

Sub-components Livelihood strategies for major component	Sub-component values for Mahe Island	Max sub-component value for study population	Min sub-component value for study population	Index value for Mahe Island	Livelihood strategies major component values for Mahe Island
% of HH dependent solely on fisheries as source of income	95	100	0	0.95	0.4
% of HH with family member employed outside the fishery sector	1.625	100	0	0.01625	
Average livelihood diversification index	0.38	1	0.2	0.225	

#### Praslin Island

**Table:**

Sub-components Livelihood strategies for major component	Sub-component values for Praslin Island	Max sub-component value for study population	Min sub-component value for study population	Index value for Praslin Island	Livelihood strategies major component values for Praslin Island
% of HH dependent solely on fisheries as source of income	97	100	0	0.97	0.38
% of HH with family member employed outside the fishery sector	0.9	100	0	0.009	
Average livelihood diversification index	0.33	1	0.2	0.1625	

### Appendix A4. Calculating the Social networks major component

#### Mahe Island

**Table:**

Sub-components Social networks for major component	Sub-component values for Mahe Island	Max sub-component value for study population	Min sub-component value for study population	Index value for Mahe Island	Social networks major component values for Mahe Island
% of HH dependent solely on fisheries as source of income	92	100	0	0.92	0.57
% of HH with family member employed outside the fishery sector	10	100	0	0.1	
Average livelihood diversification index	70	100	0	0.7	

#### Praslin Island

**Table:**

Sub-components Social networks for major component	Sub-component values for Praslin Island	Max sub-component value for study population	Min sub-component value for study population	Index value for Praslin Island	Social networks major component values for Praslin Island
% of HH dependent solely on fisheries as source of income	98	100	0	0.98	0.59
% of HH with family member employed outside the fishery sector	20	100	0	0.2	
Average livelihood diversification index	60	100	0	0.6	

### Appendix A5. Calculating the Health major component

#### Mahe Island

**Table:**

Sub-components Health for major component	Sub-component values for Mahe Island	Max sub-component value for study population	Min sub-component value for study population	Index value for Mahe Island	Social networks major component values for Mahe Island
% of HH that remains physically fit during the last 12 months to carry out livelihood activities	97	100	0	0.97	0.69
Distance to the nearest health center (Minutes)	30	60	10	0.4	

#### Praslin Island

**Table:**

Sub-components Health for major component	Sub-component values for Praslin Island	Max sub-component value for study population	Min sub-component value for study population	Index value for Praslin Island	Social networks major component values for Praslin Island
% of HH that remains physically fit during the last 12 months to carry out livelihood activities	93	100	0	0.93	0.565
Distance to the nearest health center (Minutes)	20	60	10	0.2	

### Appendix A6. Calculating the Food major component

#### Mahe Island

**Table:**

Sub-components for Food major component	Sub-component values for Mahe Island	Max sub-component value for study population	Min sub-component value for study population	Index value for Mahe Island	Food major component values for Mahe Island
% of HH that depend solely on fish/seafood in the last 12 months	20	100	0	0.2	0.373
% of heads of HH that get fish from other fishers during the last 12 months	15	100	0	0.15	
Average number of months HH struggle to find fish due to monsoon	3	5	1	0.5	
Fish diversity index	10	15	1	0.64	

#### Praslin Island

**Table:**

Sub-components for Food major component	Sub-component values for Praslin Island	Max sub-component value for study population	Min sub-component value for study population	Index value for Praslin Island	Food major component values for Praslin Island
% of HH that depend solely on fish/seafood in the last 12 months	45	100	0	0.45	0.39
% of heads of HH that get fish from other fishers during the last 12 months	50	100	0	0.5	
Average number of months HH struggle to find fish due to monsoon	2	5	1	0.25	
Fish diversity index	6	15	1	0.36	

### Appendix B1. Calculating LVI–IPCC for Mahe Island, Seychelles

**Table:**

Contributing factors	Major components for Mahe Island	Major component values for Mahe Island	Number of subcomponents per major component	Contributing factor values	LVI–IPCC value for Mahe Island
Adaptive capacity	Socio-demographic profile	0.29	4	0.407	− 0.07
	Livelihood strategies	0.4	3		
	Social networks	0.57	3		
Sensitivity	Health	0.69	2	0.479	
	Food	0.373	4		
Exposure	Natural disasters and climate variability	0.26	7	0.26	

Step 1 (calculate indexed sub-component indicators and major components as shown in Appendix A, taking the inverse of the sensitivity sub-component indicators: Social networks, Health, and food).

Step 2 (repeat for all contributing factors: exposure, sensitivity, and adaptive capacity): $\mathrm{SensitivityMahe}=\frac{{\sum}_{i=1}^{6} {W}_{{M}_{i}} {M}_{{d}_{i}}}{{\sum}_{i=1}^{6} {W}_{{M}_{i}}}=\frac{\left(2\right)\left(0.69\right)+(4)(0.373)}{2+4}=0.479$

Step 3 (repeat for all study areas): LVI—IPCC_Mahe_ = (^e^Mahe-^a^Mahe)*^s^Mahe = (0.26–0.407)*0.479 = -0.07.

### Appendix B2. Calculating LVI–IPCC for Praslin Island, Seychelles

**Table:**

Contributing factors	Major components for Praslin Island	Major component values for Praslin Island	Number of subcomponents per major component	Contributing factor values	LVI–IPCC value for Praslin Island
Adaptive capacity	Socio-demographic profile	0.21	4	0.375	0.011
	Livelihood strategies	0.38	3		
	Social networks	0.59	3		
Sensitivity	Health	0.565	2	0.448	
	Food	0.39	4		
Exposure	Natural disasters and climate variability	0.4	7	0.4	
